# 1-*tert*-Butyl 2-ethyl 5-bromo-3-(thio­phen-2-ylcarbon­yl)-1*H*-indole-1,2-dicarboxyl­ate

**DOI:** 10.1107/S1600536813000809

**Published:** 2013-01-16

**Authors:** Mohammad Hassam, Vincent J. Smith

**Affiliations:** a Department of Chemistry and Polymer Science, University of Stellenbosch, Private Bag X1, Matieland 7602, South Africa

## Abstract

In the title compound, C_21_H_20_BrNO_5_S, the thio­phene group is located above the mean plane of the indole ring and displays rotational disorder (*i.e.* rotation through 180°). The site occupancy of the major component is 0.902 (2), while that of the minor component is 0.098 (2). In the crystal, pairs of weak C—H⋯O inter­actions link the mol­ecules into centrosymmetric dimers.

## Related literature

For background to the use of indoles as scaffolds in the synthesis of HIV-agents, see: Hassam *et al.* (2012 ▶) and for a recent review on stages of non-nucleoside reverse trans­criptase inhibitors, see: Reynolds *et al.* (2012 ▶). For the crystal structures of closely related compounds, see: Beddoes *et al.* (1986 ▶), Hassam & Smith (2012 ▶).
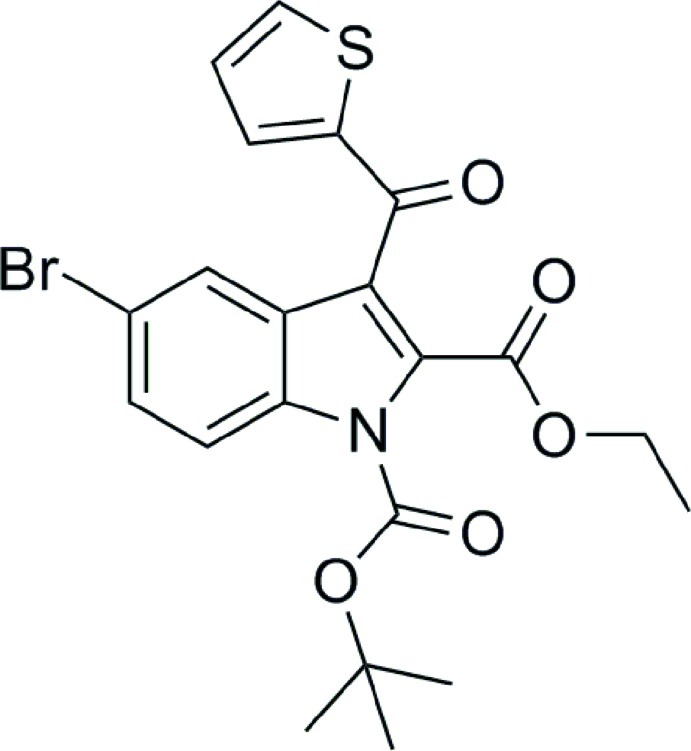


## Experimental

### 

#### Crystal data

C_21_H_20_BrNO_5_S
*M* *_r_* = 478.35
Monoclinic, 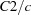

*a* = 16.220 (3) Å
*b* = 15.361 (3) Å
*c* = 18.224 (4) Å
β = 113.792 (2)°
*V* = 4154.7 (15) Å^3^
*Z* = 8
Mo *K*α radiation
μ = 2.11 mm^−1^
*T* = 100 K
0.34 × 0.21 × 0.17 mm

#### Data collection

Bruker APEXII CCD diffractometer
Absorption correction: multi-scan [symmetry-related measurements (*SADABS*; Bruker, 2009 ▶)] *T*_min_ = 0.537, *T*_max_ = 0.721
23562 measured reflections
4855 independent reflections
4101 reflections with *I* > 2σ(*I*)
*R*_int_ = 0.039

#### Refinement

*R*[*F* ^2^ > 2σ(*F* ^2^)] = 0.029
*wR*(*F* ^2^) = 0.066
*S* = 1.05
4855 reflections
279 parameters
13 restraints
H-atom parameters constrained
Δρ_max_ = 0.38 e Å^−3^
Δρ_min_ = −0.38 e Å^−3^

### 

Data collection: *APEX2* (Bruker, 2009 ▶); cell refinement: *SAINT* (Bruker, 2009 ▶); data reduction: *SAINT*; program(s) used to solve structure: *SHELXS97* (Sheldrick, 2008 ▶); program(s) used to refine structure: *SHELXL97* (Sheldrick, 2008 ▶); molecular graphics: *X-SEED* (Barbour, 2001 ▶; Atwood & Barbour, 2003 ▶); software used to prepare material for publication: *X-SEED*.

## Supplementary Material

Crystal structure: contains datablock(s) I, global. DOI: 10.1107/S1600536813000809/jj2160sup1.cif

Structure factors: contains datablock(s) I. DOI: 10.1107/S1600536813000809/jj2160Isup2.hkl

Supplementary material file. DOI: 10.1107/S1600536813000809/jj2160Isup3.cml

Additional supplementary materials:  crystallographic information; 3D view; checkCIF report

## Figures and Tables

**Table 1:** Hydrogen-bond geometry (Å, °)

*D*—H⋯*A*	*D*—H	H⋯*A*	*D*⋯*A*	*D*—H⋯*A*
C12*A*—H12*A*⋯O3^i^	0.95	2.48	3.418 (5)	169

Symmetry code: (i) 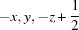.

**Fig. 1.**
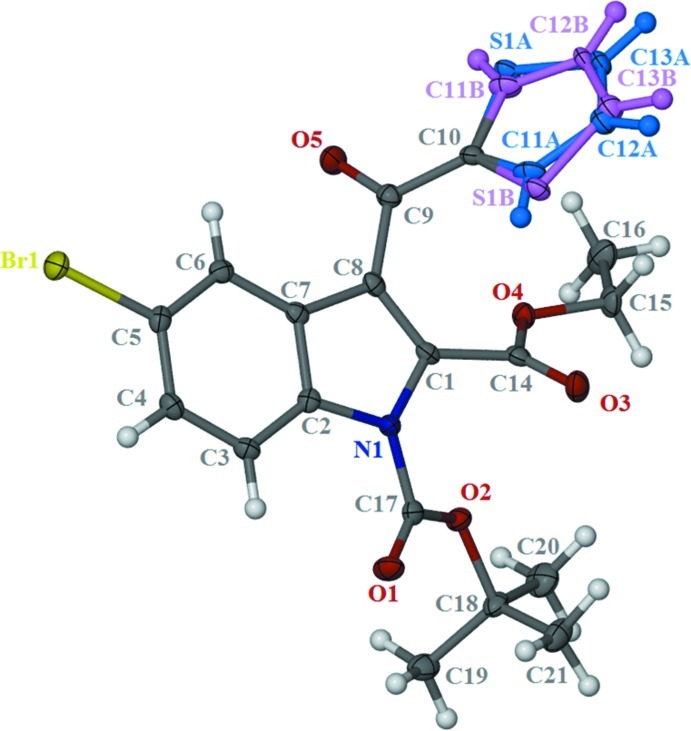
Molecular structure of the title compound with atom displacement ellipsoidsdrawn at the 50% probability level. Disordered components (0.9021 (19) = blue) and (0.0979 (19) = purple).

**Fig. 2.**
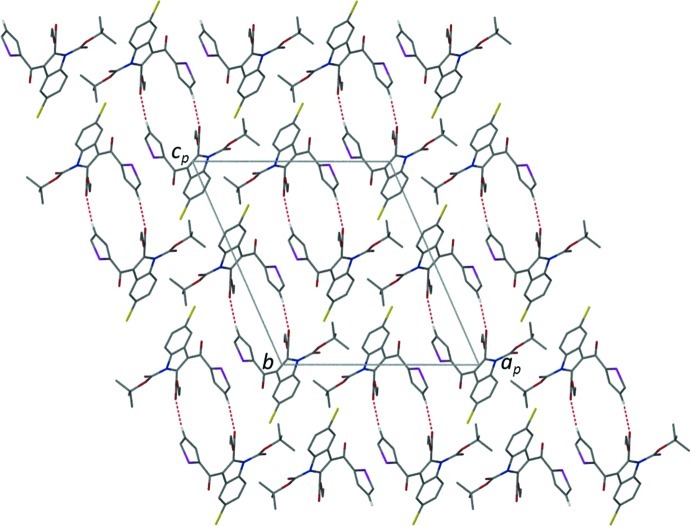
Molecular Packing of the title compound viewed along the b axis. Centrosymmetric dimers are linked via weak C—H···O intermolecular interactions (dashed lines).

## supplementary crystallographic information

### Comment

Ethyl-5-bromo-1*H*-indole-2-carboxylate has been employed as a building block in the synthesis of various anti-HIV active molecules, particularly in the search for novel non-nucleoside reverse transcriptase inhibitors (Hassam *et al.* 2012). Protection on the indole NH of ethyl 5-bromo-3-(2- thiophenoyl)-1*H*-indole-2-carboxylate was carried out with di-*tert* -butyl-dicarbonate using 4-dimethylaminopyridine as a catalytic base.

The title compound, C_21_H_20_BrNO_5_S, crystallizes with one molecule in the asymmetric unit (Fig. 1). The thiophene moiety is disordered over two positions with major (A) and minor (B) components in a 0.9021 (19):0.0979 (19)(2) ratio. The dihedral angles between the mean planes of the 5-bromo indole ring (Br1/N1/C1-C8) and the disordered thiophene rings (S1A/C10/C11A/C13A and S1B/C10/C11B/C13B) are 59.67 (9)° and 60.20 (76)°, respectively. The angles between the mean planes of the indole ring and the *N*-*tert*- butyloxy, ethyl ester and the ketone groups are 31.72 (7)°, 45.08 (6)° and 47.88 (7)°, respectively. The torsion angles of O5/C9/C10/S1A and O5/C9/C10/S1B are -20.67 (24)° and 159.92 (34)°, respectively, thereby describing the major component in a *cis* conformation and the minor component in a *trans* conformation. Molecular packing shows the molecules forming centrosymmetric dimers linked *via* weak C12A—H12A···O3 intermolecular interactions (Fig. 2, Table 1).

### Experimental

4-dimethylaminopyridine (0.0100 g, 0.0818 mmol) was added to a solution of ethyl 5-bromo-3-(2-thiophenoyl)-1*H*-indole-2-carboxylate (1.10 g, 2.91 mmol) in THF (20 ml), followed by the addition of di-*tert*-butyl dicarbonate (1.16 g, 5.32 mmol). The reaction mixture was stirred at 298.15 K for 2 h. Colourless crystals were obtained from a hexane/dichloromethane solvent mixture (4:1) (1.15 g, 83%).

### Refinement

All non-hydrogen atoms were refined anisotropically. H atoms were placed geometrically [C—H = 0.95 - 0.99 Å; with *U*_iso_(H) = 1.2 - 1.5*U*_eq_(C)] and constrained to ride on their parent atoms. The site-occupancy factors of the disordered thiophene moieties were initially set to 0.5 and then refined, leading to an occupancy of 0.9021 (19) and 0.0979 (19)(2) for the major and minor components, respectively. Bond lengths for the thiophene and phenyl moieties were restrained using the *SHELXL* SADI command (s.u. = 0.002 Å). Atom displacement parameters for overlaping atoms of the disordered models were constrained using EADP.

### Crystal data

**Table:**

C_21_H_20_BrNO_5_S	*F*(000) = 1952
*M**_r_* = 478.35	*D*_x_ = 1.530 Mg m^−^^3^
Monoclinic, *C*2/*c*	Melting point: 370.13 K
Hall symbol: -C 2yc	Mo *K*α radiation, λ = 0.71073 Å
*a* = 16.220 (3) Å	Cell parameters from 6586 reflections
*b* = 15.361 (3) Å	θ = 2.4–27.6°
*c* = 18.224 (4) Å	µ = 2.11 mm^−^^1^
β = 113.792 (2)°	*T* = 100 K
*V* = 4154.7 (15) Å^3^	Rectangular prisms, colourless
*Z* = 8	0.34 × 0.21 × 0.17 mm

### Data collection

**Table:**

Bruker APEXII CCD diffractometer	4855 independent reflections
Radiation source: fine-focus sealed tube, Bruker SMART Apex	4101 reflections with *I* > 2σ(*I*)
Graphite monochromator	*R*_int_ = 0.039
φ and ω scans	θ_max_ = 28.2°, θ_min_ = 1.9°
Absorption correction: multi-scan [symmetry-related measurements (*SADABS*; Bruker, 2009)]	*h* = −20→20
*T*_min_ = 0.537, *T*_max_ = 0.721	*k* = −19→19
23562 measured reflections	*l* = −24→24

### Refinement

**Table:**

Refinement on *F*^2^	Primary atom site location: structure-invariant direct methods
Least-squares matrix: full	Secondary atom site location: difference Fourier map
*R*[*F*^2^ > 2σ(*F*^2^)] = 0.029	Hydrogen site location: inferred from neighbouring sites
*wR*(*F*^2^) = 0.066	H-atom parameters constrained
*S* = 1.05	*w* = 1/[σ^2^(*F*_o_^2^) + (0.0296*P*)^2^ + 2.6317*P*] where *P* = (*F*_o_^2^ + 2*F*_c_^2^)/3
4855 reflections	(Δ/σ)_max_ = 0.001
279 parameters	Δρ_max_ = 0.38 e Å^−^^3^
13 restraints	Δρ_min_ = −0.38 e Å^−^^3^

### Special details

**Table:**

Geometry. All e.s.d.'s (except the e.s.d. in the dihedral angle between two l.s. planes) are estimated using the full covariance matrix. The cell e.s.d.'s are taken into account individually in the estimation of e.s.d.'s in distances, angles and torsion angles; correlations between e.s.d.'s in cell parameters are only used when they are defined by crystal symmetry. An approximate (isotropic) treatment of cell e.s.d.'s is used for estimating e.s.d.'s involving l.s. planes.
Refinement. Refinement of *F*^2^ against ALL reflections. The weighted *R*-factor *wR* and goodness of fit *S* are based on *F*^2^, conventional *R*-factors *R* are based on *F*, with *F* set to zero for negative *F*^2^. The threshold expression of *F*^2^ > σ(*F*^2^) is used only for calculating *R*-factors(gt) *etc*. and is not relevant to the choice of reflections for refinement. *R*-factors based on *F*^2^ are statistically about twice as large as those based on *F*, and *R*- factors based on ALL data will be even larger.

### Fractional atomic coordinates and isotropic or equivalent isotropic displacement parameters (Å^2^)

**Table:**

	*x*	*y*	*z*	*U*_iso_*/*U*_eq_	Occ. (<1)
Br1	−0.187401 (12)	−0.022969 (12)	−0.284234 (11)	0.02154 (6)	
O1	0.21832 (9)	0.04217 (8)	0.06003 (8)	0.0220 (3)	
N1	0.09114 (9)	0.12572 (9)	0.02039 (8)	0.0140 (3)	
C1	0.04334 (12)	0.19832 (11)	0.02894 (10)	0.0143 (4)	
O2	0.22623 (8)	0.18743 (8)	0.08505 (7)	0.0168 (3)	
C2	0.03540 (10)	0.08118 (9)	−0.04949 (10)	0.0143 (3)	
O3	0.11295 (9)	0.22588 (8)	0.16936 (7)	0.0223 (3)	
C3	0.05264 (11)	0.00587 (10)	−0.08360 (9)	0.0165 (4)	
H3	0.1083	−0.0243	−0.0598	0.020*	
O4	0.06607 (8)	0.33759 (8)	0.08185 (7)	0.0167 (3)	
C4	−0.01584 (11)	−0.02275 (11)	−0.15432 (10)	0.0173 (4)	
H4	−0.0072	−0.0740	−0.1795	0.021*	
O5	−0.14693 (9)	0.30149 (9)	−0.11372 (7)	0.0229 (3)	
C5	−0.09682 (11)	0.02238 (10)	−0.18879 (10)	0.0167 (4)	
C6	−0.11345 (11)	0.09814 (10)	−0.15555 (9)	0.0159 (4)	
H6	−0.1687	0.1289	−0.1802	0.019*	
C7	−0.04550 (10)	0.12713 (11)	−0.08437 (9)	0.0143 (3)	
C8	−0.03952 (12)	0.20088 (11)	−0.03284 (10)	0.0147 (4)	
C9	−0.11022 (12)	0.26847 (12)	−0.04742 (11)	0.0165 (4)	
C10	−0.13346 (11)	0.29232 (11)	0.01992 (10)	0.0147 (4)	
C14	0.07984 (11)	0.25376 (11)	0.10226 (11)	0.0158 (4)	
C15	0.08485 (13)	0.39806 (12)	0.14842 (11)	0.0217 (4)	
H15A	0.1485	0.3932	0.1869	0.026*	
H15B	0.0457	0.3853	0.1771	0.026*	
C16	0.06589 (15)	0.48791 (13)	0.11250 (13)	0.0294 (5)	
H16C	0.0026	0.4918	0.0748	0.044*	
H16A	0.1047	0.4994	0.0840	0.044*	
H16B	0.0780	0.5310	0.1553	0.044*	
C17	0.18541 (12)	0.11267 (11)	0.05822 (10)	0.0159 (4)	
C18	0.32643 (12)	0.19106 (12)	0.13180 (11)	0.0190 (4)	
C19	0.37308 (13)	0.16063 (14)	0.07930 (12)	0.0249 (4)	
H19C	0.3471	0.1907	0.0275	0.037*	
H19A	0.3648	0.0977	0.0708	0.037*	
H19B	0.4376	0.1738	0.1056	0.037*	
C20	0.34088 (14)	0.28765 (13)	0.14976 (13)	0.0293 (5)	
H20A	0.3061	0.3063	0.1802	0.044*	
H20B	0.3209	0.3202	0.0993	0.044*	
H20C	0.4050	0.2988	0.1814	0.044*	
C21	0.35137 (14)	0.13828 (14)	0.20765 (11)	0.0278 (5)	
H21A	0.3417	0.0763	0.1940	0.042*	
H21C	0.3137	0.1561	0.2357	0.042*	
H21B	0.4149	0.1481	0.2425	0.042*	
S1A	−0.18632 (4)	0.39061 (3)	0.01797 (3)	0.01650 (16)	0.9021 (19)
C11A	−0.12031 (18)	0.24830 (18)	0.08752 (18)	0.0212 (5)	0.902 (2)
H11A	−0.0924	0.1927	0.0987	0.025*	0.9021 (19)
C12A	−0.1506 (2)	0.2902 (3)	0.1402 (3)	0.0196 (4)	0.9021 (19)
H12A	−0.1462	0.2667	0.1899	0.024*	0.9021 (19)
C13A	−0.1874 (3)	0.3695 (3)	0.11084 (15)	0.0161 (6)	0.9021 (19)
H13A	−0.2108	0.4084	0.1382	0.019*	0.9021 (19)
S1B	−0.1088 (5)	0.2256 (4)	0.1026 (3)	0.01650 (16)	0.0979 (19)
C11B	−0.1782 (13)	0.3643 (9)	0.0245 (15)	0.0212 (5)	0.0979 (19)
H11B	−0.1971	0.4082	−0.0157	0.025*	0.0979 (19)
C12B	−0.194 (3)	0.368 (3)	0.0944 (18)	0.0161 (6)	0.0979 (19)
H12B	−0.2251	0.4149	0.1065	0.019*	0.0979 (19)
C13B	−0.161 (2)	0.298 (2)	0.143 (3)	0.0196 (4)	0.0979 (19)
H13B	−0.1655	0.2895	0.1930	0.024*	0.0979 (19)

### Atomic displacement parameters (Å^2^)

**Table:**

	*U*^11^	*U*^22^	*U*^33^	*U*^12^	*U*^13^	*U*^23^
Br1	0.01920 (10)	0.02324 (11)	0.01899 (10)	−0.00231 (8)	0.00438 (7)	−0.00483 (7)
O1	0.0186 (7)	0.0174 (7)	0.0266 (7)	0.0045 (5)	0.0056 (6)	−0.0011 (5)
N1	0.0133 (7)	0.0144 (7)	0.0138 (7)	0.0008 (6)	0.0051 (6)	−0.0017 (6)
C1	0.0166 (9)	0.0130 (8)	0.0164 (9)	0.0025 (7)	0.0099 (7)	0.0014 (7)
O2	0.0136 (6)	0.0167 (6)	0.0178 (6)	−0.0006 (5)	0.0040 (5)	−0.0017 (5)
C2	0.0147 (8)	0.0139 (8)	0.0145 (8)	−0.0028 (7)	0.0061 (7)	0.0000 (7)
O3	0.0296 (7)	0.0216 (7)	0.0148 (7)	0.0044 (6)	0.0078 (6)	0.0005 (5)
C3	0.0165 (9)	0.0145 (9)	0.0199 (9)	0.0022 (7)	0.0087 (7)	0.0006 (7)
O4	0.0196 (6)	0.0139 (6)	0.0158 (6)	−0.0004 (5)	0.0062 (5)	−0.0028 (5)
C4	0.0213 (9)	0.0141 (8)	0.0189 (9)	−0.0002 (7)	0.0106 (8)	−0.0022 (7)
O5	0.0266 (7)	0.0251 (7)	0.0167 (7)	0.0076 (6)	0.0084 (6)	0.0043 (5)
C5	0.0176 (9)	0.0186 (9)	0.0138 (8)	−0.0040 (7)	0.0065 (7)	−0.0013 (7)
C6	0.0152 (9)	0.0179 (9)	0.0158 (9)	0.0003 (7)	0.0075 (7)	0.0019 (7)
C7	0.0158 (9)	0.0143 (8)	0.0158 (9)	0.0009 (7)	0.0093 (7)	0.0016 (7)
C8	0.0185 (9)	0.0143 (8)	0.0146 (9)	0.0008 (7)	0.0103 (7)	0.0010 (7)
C9	0.0154 (9)	0.0165 (9)	0.0183 (9)	−0.0005 (7)	0.0074 (7)	−0.0011 (7)
C10	0.0116 (8)	0.0154 (8)	0.0172 (9)	0.0019 (7)	0.0059 (7)	−0.0003 (7)
C14	0.0131 (8)	0.0175 (9)	0.0193 (9)	0.0013 (7)	0.0091 (7)	−0.0015 (7)
C15	0.0258 (10)	0.0202 (10)	0.0167 (9)	−0.0030 (8)	0.0060 (8)	−0.0082 (7)
C16	0.0334 (12)	0.0202 (10)	0.0264 (11)	0.0014 (9)	0.0034 (9)	−0.0064 (8)
C17	0.0172 (9)	0.0186 (9)	0.0124 (8)	0.0006 (7)	0.0068 (7)	0.0005 (7)
C18	0.0128 (8)	0.0260 (10)	0.0161 (9)	−0.0030 (7)	0.0036 (7)	−0.0009 (7)
C19	0.0187 (10)	0.0322 (11)	0.0250 (10)	0.0017 (8)	0.0100 (8)	0.0047 (9)
C20	0.0249 (11)	0.0277 (11)	0.0309 (11)	−0.0083 (9)	0.0068 (9)	−0.0066 (9)
C21	0.0235 (10)	0.0381 (12)	0.0178 (10)	−0.0044 (9)	0.0043 (8)	0.0046 (9)
S1A	0.0180 (3)	0.0143 (3)	0.0192 (3)	0.0051 (2)	0.0095 (2)	0.0010 (2)
C11A	0.0184 (13)	0.0143 (13)	0.0291 (14)	0.0058 (10)	0.0080 (11)	0.0014 (10)
C12A	0.0206 (13)	0.0209 (13)	0.0189 (10)	0.0016 (10)	0.0096 (10)	0.0000 (8)
C13A	0.0188 (12)	0.0188 (9)	0.0142 (15)	0.0031 (9)	0.0102 (14)	−0.0018 (14)
S1B	0.0180 (3)	0.0143 (3)	0.0192 (3)	0.0051 (2)	0.0095 (2)	0.0010 (2)
C11B	0.0184 (13)	0.0143 (13)	0.0291 (14)	0.0058 (10)	0.0080 (11)	0.0014 (10)
C12B	0.0188 (12)	0.0188 (9)	0.0142 (15)	0.0031 (9)	0.0102 (14)	−0.0018 (14)
C13B	0.0206 (13)	0.0209 (13)	0.0189 (10)	0.0016 (10)	0.0096 (10)	0.0000 (8)

### Geometric parameters (Å, º)

**Table:**

Br1—C5	1.8993 (17)	C15—H15A	0.9900
O1—C17	1.202 (2)	C15—H15B	0.9900
N1—C1	1.402 (2)	C16—H16C	0.9800
N1—C2	1.406 (2)	C16—H16A	0.9800
N1—C17	1.415 (2)	C16—H16B	0.9800
C1—C8	1.362 (2)	C18—C21	1.510 (3)
C1—C14	1.491 (2)	C18—C19	1.514 (3)
O2—C17	1.317 (2)	C18—C20	1.517 (3)
O2—C18	1.501 (2)	C19—H19C	0.9800
C2—C3	1.3940 (15)	C19—H19A	0.9800
C2—C7	1.3968 (15)	C19—H19B	0.9800
O3—C14	1.199 (2)	C20—H20A	0.9800
C3—C4	1.3903 (16)	C20—H20B	0.9800
C3—H3	0.9500	C20—H20C	0.9800
O4—C14	1.334 (2)	C21—H21A	0.9800
O4—C15	1.459 (2)	C21—H21C	0.9800
C4—C5	1.3909 (16)	C21—H21B	0.9800
C4—H4	0.9500	S1A—C13A	1.730 (3)
O5—C9	1.221 (2)	C11A—C12A	1.400 (3)
C5—C6	1.3878 (15)	C11A—H11A	0.9500
C6—C7	1.3935 (16)	C12A—C13A	1.367 (3)
C6—H6	0.9500	C12A—H12A	0.9500
C7—C8	1.450 (2)	C13A—H13A	0.9500
C8—C9	1.489 (2)	S1B—C13B	1.730 (3)
C9—C10	1.468 (2)	C11B—C12B	1.400 (4)
C10—C11B	1.344 (4)	C11B—H11B	0.9500
C10—C11A	1.345 (3)	C12B—C13B	1.367 (3)
C10—S1A	1.7297 (17)	C12B—H12B	0.9500
C10—S1B	1.730 (2)	C13B—H13B	0.9500
C15—C16	1.505 (3)		
			
C1—N1—C2	107.77 (13)	H16C—C16—H16A	109.5
C1—N1—C17	126.56 (15)	C15—C16—H16B	109.5
C2—N1—C17	123.07 (13)	H16C—C16—H16B	109.5
C8—C1—N1	109.67 (15)	H16A—C16—H16B	109.5
C8—C1—C14	128.83 (16)	O1—C17—O2	128.62 (17)
N1—C1—C14	121.09 (15)	O1—C17—N1	121.44 (16)
C17—O2—C18	120.67 (14)	O2—C17—N1	109.86 (15)
C3—C2—C7	122.48 (14)	O2—C18—C21	109.18 (15)
C3—C2—N1	129.51 (13)	O2—C18—C19	109.43 (14)
C7—C2—N1	108.00 (12)	C21—C18—C19	113.28 (17)
C4—C3—C2	116.55 (15)	O2—C18—C20	101.33 (14)
C4—C3—H3	121.7	C21—C18—C20	111.51 (16)
C2—C3—H3	121.7	C19—C18—C20	111.42 (16)
C14—O4—C15	115.31 (14)	C18—C19—H19C	109.5
C3—C4—C5	121.11 (15)	C18—C19—H19A	109.5
C3—C4—H4	119.4	H19C—C19—H19A	109.5
C5—C4—H4	119.4	C18—C19—H19B	109.5
C6—C5—C4	122.36 (15)	H19C—C19—H19B	109.5
C6—C5—Br1	119.51 (11)	H19A—C19—H19B	109.5
C4—C5—Br1	118.13 (11)	C18—C20—H20A	109.5
C5—C6—C7	117.02 (15)	C18—C20—H20B	109.5
C5—C6—H6	121.5	H20A—C20—H20B	109.5
C7—C6—H6	121.5	C18—C20—H20C	109.5
C6—C7—C2	120.47 (14)	H20A—C20—H20C	109.5
C6—C7—C8	132.37 (15)	H20B—C20—H20C	109.5
C2—C7—C8	107.16 (14)	C18—C21—H21A	109.5
C1—C8—C7	107.38 (15)	C18—C21—H21C	109.5
C1—C8—C9	126.85 (16)	H21A—C21—H21C	109.5
C7—C8—C9	125.72 (15)	C18—C21—H21B	109.5
O5—C9—C10	122.43 (16)	H21A—C21—H21B	109.5
O5—C9—C8	119.94 (16)	H21C—C21—H21B	109.5
C10—C9—C8	117.62 (15)	C10—S1A—C13A	90.96 (18)
C11B—C10—C11A	104.3 (12)	C10—C11A—C12A	115.3 (3)
C11B—C10—C9	125.8 (12)	C10—C11A—H11A	122.3
C11A—C10—C9	129.86 (16)	C12A—C11A—H11A	122.3
C11A—C10—S1A	110.60 (15)	C13A—C12A—C11A	111.1 (4)
C9—C10—S1A	119.53 (12)	C13A—C12A—H12A	124.5
C11B—C10—S1B	112.0 (12)	C11A—C12A—H12A	124.5
C9—C10—S1B	122.2 (3)	C12A—C13A—S1A	112.1 (4)
S1A—C10—S1B	118.3 (3)	C12A—C13A—H13A	124.0
O3—C14—O4	125.80 (16)	S1A—C13A—H13A	124.0
O3—C14—C1	124.23 (16)	C13B—S1B—C10	90.7 (19)
O4—C14—C1	109.90 (15)	C10—C11B—C12B	113 (3)
O4—C15—C16	106.54 (15)	C10—C11B—H11B	123.4
O4—C15—H15A	110.4	C12B—C11B—H11B	123.4
C16—C15—H15A	110.4	C13B—C12B—C11B	113 (4)
O4—C15—H15B	110.4	C13B—C12B—H12B	123.6
C16—C15—H15B	110.4	C11B—C12B—H12B	123.6
H15A—C15—H15B	108.6	C12B—C13B—S1B	111 (4)
C15—C16—H16C	109.5	C12B—C13B—H13B	124.4
C15—C16—H16A	109.5	S1B—C13B—H13B	124.4
			
C2—N1—C1—C8	−0.25 (19)	C8—C9—C10—S1B	−19.6 (4)
C17—N1—C1—C8	161.74 (16)	C15—O4—C14—O3	−6.9 (2)
C2—N1—C1—C14	173.05 (15)	C15—O4—C14—C1	170.13 (14)
C17—N1—C1—C14	−25.0 (3)	C8—C1—C14—O3	127.0 (2)
C1—N1—C2—C3	179.52 (17)	N1—C1—C14—O3	−44.9 (3)
C17—N1—C2—C3	16.8 (3)	C8—C1—C14—O4	−50.1 (2)
C1—N1—C2—C7	1.06 (19)	N1—C1—C14—O4	138.06 (16)
C17—N1—C2—C7	−161.70 (15)	C14—O4—C15—C16	179.51 (15)
C7—C2—C3—C4	−1.0 (3)	C18—O2—C17—O1	−6.5 (3)
N1—C2—C3—C4	−179.25 (17)	C18—O2—C17—N1	176.60 (14)
C2—C3—C4—C5	0.3 (3)	C1—N1—C17—O1	165.06 (17)
C3—C4—C5—C6	0.7 (3)	C2—N1—C17—O1	−35.5 (3)
C3—C4—C5—Br1	−178.72 (14)	C1—N1—C17—O2	−17.8 (2)
C4—C5—C6—C7	−1.1 (3)	C2—N1—C17—O2	141.64 (15)
Br1—C5—C6—C7	178.34 (12)	C17—O2—C18—C21	−61.6 (2)
C5—C6—C7—C2	0.4 (2)	C17—O2—C18—C19	62.9 (2)
C5—C6—C7—C8	−178.75 (17)	C17—O2—C18—C20	−179.30 (15)
C3—C2—C7—C6	0.6 (3)	C11B—C10—S1A—C13A	24 (8)
N1—C2—C7—C6	179.21 (15)	C11A—C10—S1A—C13A	0.89 (19)
C3—C2—C7—C8	179.98 (16)	C9—C10—S1A—C13A	−179.27 (18)
N1—C2—C7—C8	−1.43 (19)	S1B—C10—S1A—C13A	0.2 (3)
N1—C1—C8—C7	−0.63 (19)	C11B—C10—C11A—C12A	−3.2 (11)
C14—C1—C8—C7	−173.26 (16)	C9—C10—C11A—C12A	179.8 (2)
N1—C1—C8—C9	−178.36 (16)	S1A—C10—C11A—C12A	−0.4 (3)
C14—C1—C8—C9	9.0 (3)	S1B—C10—C11A—C12A	175 (3)
C6—C7—C8—C1	−179.46 (18)	C10—C11A—C12A—C13A	−0.5 (3)
C2—C7—C8—C1	1.28 (19)	C11A—C12A—C13A—S1A	1.2 (2)
C6—C7—C8—C9	−1.7 (3)	C10—S1A—C13A—C12A	−1.20 (17)
C2—C7—C8—C9	179.04 (16)	C11B—C10—S1B—C13B	0.0 (4)
C1—C8—C9—O5	131.0 (2)	C11A—C10—S1B—C13B	−2 (3)
C7—C8—C9—O5	−46.3 (3)	C9—C10—S1B—C13B	−177.5 (11)
C1—C8—C9—C10	−49.4 (3)	S1A—C10—S1B—C13B	3.1 (10)
C7—C8—C9—C10	133.27 (18)	C11A—C10—C11B—C12B	0.3 (6)
O5—C9—C10—C11B	−17.3 (12)	C9—C10—C11B—C12B	177.4 (11)
C8—C9—C10—C11B	163.2 (11)	S1A—C10—C11B—C12B	−157 (8)
O5—C9—C10—C11A	159.1 (2)	S1B—C10—C11B—C12B	0.0 (5)
C8—C9—C10—C11A	−20.4 (3)	C10—C11B—C12B—C13B	0.0 (3)
O5—C9—C10—S1A	−20.7 (2)	C11B—C12B—C13B—S1B	0.0 (2)
C8—C9—C10—S1A	159.78 (13)	C10—S1B—C13B—C12B	0.0 (3)
O5—C9—C10—S1B	159.9 (3)		

### Hydrogen-bond geometry (Å, º)

**Table:**

*D*—H···*A*	*D*—H	H···*A*	*D*···*A*	*D*—H···*A*
C12*A*—H12*A*···O3^i^	0.95	2.48	3.418 (5)	169

Symmetry code: (i) −*x*, *y*, −*z*+1/2.
